# 622. Development of Interferon-Gamma Release Assays for Diagnosing Latent Talaromycosis

**DOI:** 10.1093/ofid/ofad500.688

**Published:** 2023-11-27

**Authors:** Helen Xu, Thu Nguyen, Sruthi Venugopalan, Vo Trieu Ly, Ngo Thi Hoa, Jian-Piao Cai, Jasper F W Chan, Kwok-Yung Yuen, Thuy Le

**Affiliations:** Duke University, Durham, North Carolina; Duke University School of Medicine, Durham, North Carolina; Duke University School of Medicine, Durham, North Carolina; University of medicine and pharmacy at Ho Chi Minh city, Vietnam, Ho Chi Minh, Ho Chi Minh, Vietnam; OUCRU, Ho Chi Minh City, Ho Chi Minh, Vietnam; The University of Hong Kong, HONG KONG, Not Applicable, Hong Kong; The University of Hong Kong, HONG KONG, Not Applicable, Hong Kong; The University of Hong Kong, HONG KONG, Not Applicable, Hong Kong; Duke University School of Medicine, Durham, North Carolina

## Abstract

**Background:**

*Talaromyces marneffei* (Tm), a fungus endemic in Southeast Asia, is a leading cause of death in patients with advanced HIV disease. Existing pathogen-based assays can only detect Tm disease during advanced stages when treatment is least effective. Host-based assays could detect clinically-silent infection, allowing prophylaxis therapy to prevent disease development. Here, we report the development of novel interferon gamma (IFN-γ) release assays (IGRAs) to diagnose latent Tm infection.

**Methods:**

Tm IGRAs exploit host memory T cells’ ability to recall Tm exposure and produce IFN-γ upon stimulation with a Tm*-*specific recombinant mannoprotein Mp1p (rMp1p). We employed a case-control study design. Cases were HIV-infected subjects with a history of culture-confirmed Tm infection (N=8). Controls were healthy volunteers who have never traveled to Southeast Asia (N=7). We measured IFN-γ release from: 1) rMp1p-stimulated whole blood using the enzyme linked immunosorbent assay (ELISA) and 2) rMp1p-stimulated peripheral blood mononuclear cells (PBMCs) using the enzyme-linked immunosorbent spot (ELISpot) method. We optimized assay conditions, determined cutoff points to differentiate between cases and controls, and estimated assay sensitivity and specificity.

Tm interferon-gamma release assay principle

**Figure:**
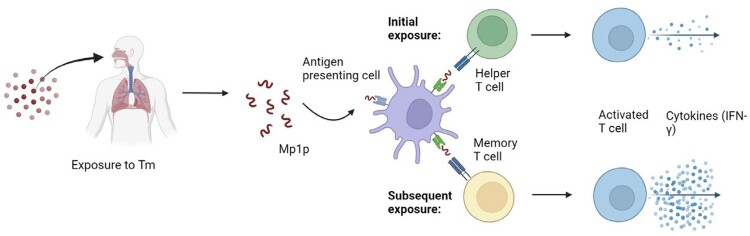

rMp1p, via antigen presenting cells, elicits a Tm-specific T-cell response with IFN-γ release. Higher levels of IFN-γ release during subsequent exposure to Tm (compared to the initial exposure) can be used to differentiate between exposed (latent or active infections) versus unexposed (naïve) individuals.

IFN-γ release measured by the ELISA and ELISpot methods

**Figure:**
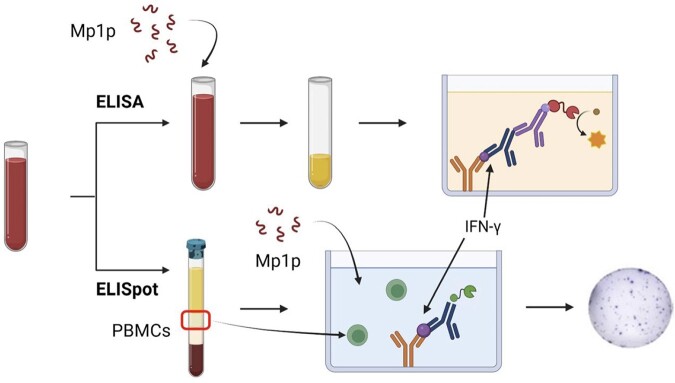

We utilize two methods to measure IFN-γ release: enzyme-linked immunosorbent assay (ELISA) and enzyme-linked immunosorbent spot (ELISpot) assay. For the ELISA, whole blood samples are stimulated with rMp1p, and IFN-γ present in the plasma is measured by an indirect sandwich ELISA method with optical density (OD) as the measurable output. For the ELISpot method, peripheral blood mononuclear cells (PBMCs) are isolated from whole blood and incubated with rMp1p. IFN-γ is captured by capture antibodies and sandwiched by biotinylated detection antibodies. Streptavidin HRP conjugate is then added. Since a substrate with a precipitating product is used, the result is visible spots on the surface of the well, where each spot corresponds to an individual cytokine-secreting cell.

**Results:**

For the ELISA-based IGRA, 1 mL of whole blood, 40 μg/mL of rMp1p, and 24-hour incubation produced the highest analytical sensitivity and specificity. The difference in mean optical density (OD) between cases and controls of 0.49 ± 0.17 was statistically significant (*P*=0.01, T-test). At the optimal OD cutoff of 0.12, the ELISA-based IGRA has 87.5% clinical sensitivity and 100% specificity. For the ELISpot-based IGRA, 5x10^5^ PBMCs, 40 μg/mL of rMp1p, and 40-hour incubation was optimal. The difference in mean spot forming units (SFUs) between cases and controls of 88 was statistically significant (*P*=0.001, T-test). At the optimal SFU cutoff of 18, the ELISpot-based IGRA has 100% clinical sensitivity and specificity.

Diagnostic performance of the ELISA-based IGRA

**Figure:**
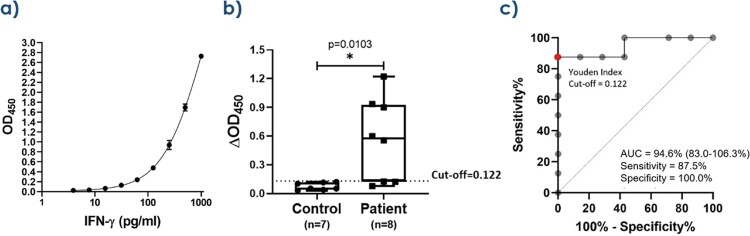

a) Standard curve showing optical density (OD) values for known concentrations of IFN-γ. The standard curve is used to determine the IFN-γ concentration of patient samples through interpolation. b) Distribution of OD values between 7 controls and 8 cases. The difference in mean OD values between cases and controls is 0.49±0.17, P=0.01 (T-test). c) The receiver operating characteristic (ROC) curve demonstrates excellent discrimination between cases and controls with 94.6% accuracy. The OD cutoff of 0.12 is the Youden index calculated from the ROC curve, which maximizes true positives, minimizes false positives, and yields a preliminary clinical sensitivity of 87.5% (95% CI: 47.4% to 99.7%) and a specificity of 100% (95% CI: 59.0% to 100.0%).

Diagnostic performance of the ELISpot-based IGRA

**Figure:**
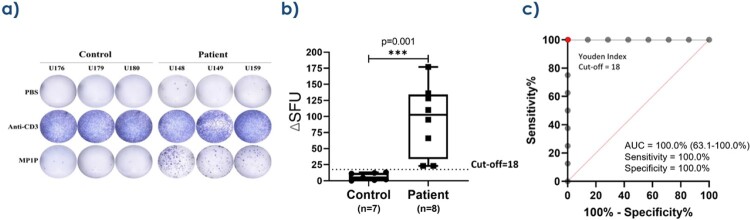

a) ELISpot results demonstrating IFN-γ release for 3 controls and 3 case patients when stimulated with PBS (negative control) in row 1, with anti-CD3 (positive control) in row 2, and with rMp1p (producing differential IFN-γ levels) in row 3. b) Distribution of spot forming units (SFUs) between 7 controls and 8 cases. The mean SFUs is 6.3 for controls and 94.8 for cases. The difference in mean SFUs between cases and controls of 88±21 is statistically significant (P=0.001, T-test). c) The receiver operating characteristic (ROC) curve demonstrates excellent discrimination between cases and controls with 100% accuracy. The SFU cutoff of 18 is the Youden index calculated from the ROC curve, which maximizes true positives, minimizes false positives, and yields a preliminary clinical sensitivity of 100% (95% CI: 63.1% to 100.0%) and a specificity of 100% (95% CI: 59.0% to 100.0%).

**Conclusion:**

This proof-of-concept study demonstrates that rMp1p elicits a robust memory T-cell response to Tm in humans. The ELISA-based and ELISpot-based IGRAs have promising clinical performance and are being further developed for testing in clinical populations.

**Disclosures:**

**Jasper FW Chan, MBBS(HK), MD(HK), FIDSA**, IMMY: Development of Talaromyces diagnostics

